# Targeting Glioblastoma Cells Expressing CD44 with Liposomes Encapsulating Doxorubicin and Displaying Chlorotoxin-IgG Fc Fusion Protein

**DOI:** 10.3390/ijms19030659

**Published:** 2018-02-26

**Authors:** Hafizah Mahmud, Tomonari Kasai, Apriliana Cahya Khayrani, Mami Asakura, Aung Ko Ko Oo, Juan Du, Arun Vaidyanath, Samah El-Ghlban, Akifumi Mizutani, Akimasa Seno, Hiroshi Murakami, Junko Masuda, Masaharu Seno

**Affiliations:** 1 Department of Medical Bioengineering, Graduate School of Natural Science and Technology, Okayama University, Okayama 700-0080, Japan; hfzhmhmd@gmail.com (H.M.); t-kasai@okayama-u.ac.jp (T.K.); apriliana41@gmail.com (A.C.K.); asakura@okayama-u.ac.jp (M.A.); kokooo.aung@gmail.com (A.K.K.O.); djmail@yeah.net (J.D.); arunvnath@okayama-u.ac.jp (A.V.); mizut-a@cc.okayama-u.ac.jp (A.M.); aseno@okayama-u.ac.jp (A.S.); muraka-h@cc.okayama-u.ac.jp (H.M.); junkomasuda@okayama-u.ac.jp (J.M.); 2 Department of Chemistry, Biochemistry Division, Faculty of Science, El Menoufia University, Shebin El Kom, Menoufia 32511, Egypt; S_elghlban@yahoo.com

**Keywords:** M-CTX-Fc, liposome, glioblastoma cells, specific targeting, doxorubicin, MMP-2

## Abstract

We recently have established a successful xenograft model of human glioblastoma cells by enriching hyaluronic acid-dependent spheroid-forming populations termed U251MG-P1 cells from U251MG cells. Since U251MG-P1 cells have been confirmed to express CD44 along with principal stemness marker genes, *OCT3/4*, *SOX2*, *KLF4* and *Nanog*, this CD44 expressing population appeared to majorly consist of undifferentiated cells. Evaluating the sensitivity to anti-cancer agents, we found U251MG-P1 cells were sensitive to doxorubicin with IC_50_ at 200 nM. Although doxorubicin has serious side-effects, establishment of an efficient therapy targeting undifferentiated glioblastoma cell population is necessary. We previously designed a chlorotoxin peptide fused to human IgG Fc region without hinge sequence (M-CTX-Fc), which exhibited a stronger growth inhibitory effect on the glioblastoma cell line A172 than an original chlorotoxin peptide. Combining these results together, we designed M-CTX-Fc conjugated liposomes encapsulating doxorubicin and used U251MG-P1 cells as the target model in this study. The liposome modified with M-CTX-Fc was designed with a diameter of approximately 100–150 nm and showed high encapsulation efficiency, adequate loading capacity of anticancer drug, enhanced antitumor effects demonstrating increasing uptake into the cells in vitro; M-CTX-Fc-L-Dox shows great promise in its ability to suppress tumor growth in vivo and it could serve as a template for targeted delivery of other therapeutics.

## 1. Introduction

Glioblastoma is a highly invasive cancer where the cells demonstrate their infiltrative growth to diffuse into the brain tissue [1]. This is the reason why the treatment of glioblastoma requires a multidisciplinary approach. Current standard of care for glioblastoma includes maximal safe surgical resection followed by radiotherapy and chemotherapy with the alkylating agent Temozolomide. The extensive and complete surgical treatment involves difficulties in glioblastoma with high degree of invasion because simultaneous removal of the surrounding normal areas will impair the function of brain controlling speech, motor function, sense and personality [2]. On that occasion, developing of brain tumor specific targeting drug delivery systems, which increase drug accumulation in the tumor region with less toxicity to the adjacent normal brain tissue, would significantly be a great approach for brain tumor treatments. The drug delivery to brain faces has been considered difficult as the agents need to across the blood–brain barrier (BBB). However, a recent study has shown that the tight junction of the BBB loses the integrity by increasing permeability of the capillary endothelium [3].

On the other hand, recurrence of relapsing cancer is currently the central big issue to be studied. This is considered to occur due to the residual subpopulation of cancer cells after the treatment because they are believed to be resistant to chemotherapy and radiotherapy even though they are the minor population in the cancer tissues [4]. In this context, chemical agents toxically effective against cancer cells should properly be chosen to design an effective drug delivery system.

As for the efficacy of drug delivery, targeting ability is another strong issue to be designed. Some cell surface antigens including receptors specific to gliomas and/or neovasculatures will be crucial markers to be targeted [5]. Hence, several approaches to treat brain cancer employ ligands specific to the tumor cells targeting cell surface markers, which are overexpressed in cancer cells but low or not expressed in normal cells.

Matrix metalloproteinase-2 (MMP-2) is an extracellular matrix degrading enzyme, which plays an important role in tumor invasion and is highly expressed in related cancer cells [6]. As for MMP-2 in glioblastoma, the activity is increased along with the tumor grade and the expression is significantly higher than that in normal brain tissue. The increment is associated with poor prognosis and the overall short-life of survivors. MMP-2 is secreted as an inactive zymogen and prior to its activation, it will bind to tissue inhibitor of metalloproteinase-2 associated with membrane type matrix metalloproteinase-1, which localizes on the cell surface of the tumors [7,8]. Membrane type matrix metalloproteinase-1 is replenished by auto degradation or clathrin-dependent internalization. Collectively, MMP-2 has been considered as a target for cancer therapy.

Chlorotoxin (CTX) is a peptide derived from Egyptian scorpion venom, which has initially been characterized as an MMP-2 inhibitor and also as a voltage-gated chloride channel blocker [9,10]. CTX exhibited high specificity, selectivity and affinity for glioma and other tumors of neuroectodermal origin [11]. Following the discovery, CTX has been extensively developed as a ligand for active targeting to deliver cytotoxic agents, fluorescent dyes for imaging and iodine for labeling tumor cells [12,13,14]. Recent findings suggested that the delivery of CTX-modified liposomes was mediated by MMP-2 but not correlated with the chloride channel CIC-3 when targeting U87 glioma cells [15].

In our previous report, the CTX fused to human IgG Fc domain without hinge region in monomeric form (M-CTX-Fc) showed inhibition of mortality of the glioma cell line A172 [16]. This inhibitory effect was enhanced when compared to the original CTX peptide. The similar effect was observed in pancreatic cancer cell PANC-1 cells [17]. Indicating M-CTX-Fc could be a potential ligand for active targeting of glioblastoma cells, the target-dependent internalization of bionanocapsules displaying M-CTX-Fc on the surface into cells was described [16]. 

Very recently, we condensed the population overexpressing CD44 in U251MG cells exploiting the preferential affinity for hyaluronic acid as U251MG-P1 cells [18]. Since CD44 is well known as a common marker of cancer stem cells, we confirmed the expression of stemness markers as well as the tumor-initiating capacity in U251MG-P1 cells.

In this study, we designed a liposomal drug delivery system of doxorubicin to evaluate the ability of M-CTX-Fc as an effective moiety to target glioblastoma cells expressing stemness markers using U251MG-P1 cells as the target model.

## 2. Results and Discussion

### 2.1. Sensitivity of U251MG-P1 Cells to Doxorubicin

**Figure 1:**
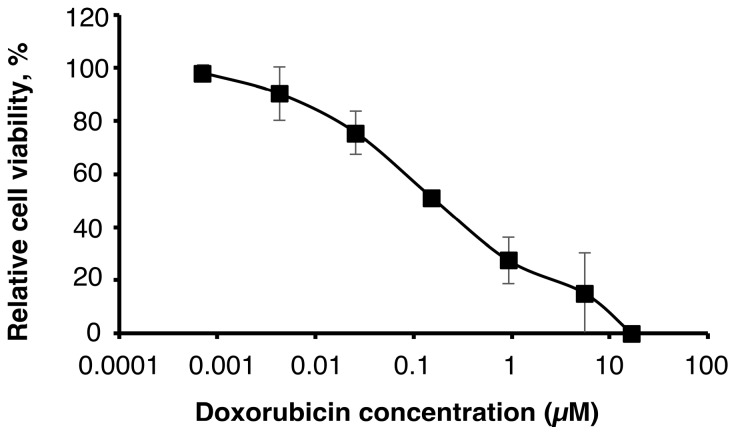
The U251MG-P1 cells are sensitive to doxorubicin. Cytotoxicity of doxorubicin was assessed on U251MG-P1 cells by MTT assay. The data presented as the mean ± SD (*n* = 3) from independent experiment.

First of all, we assessed the effect of doxorubicin on U251MG-P1 cells (Figure 1). We chose doxorubicin as the first priority because the liposomal formulation is well established by pH gradient and ammonium sulfate gradient method and has clinically been tested in glioblastoma cancer therapy [19,20,21,22]. As a result, the MTT assay showed an IC_50_ at around 200 nM, which was a feasible concentration available as an agent for cancer chemotherapy. 

However, cardiotoxicity is the well-known side effect of doxorubicin, so that the amount of administration through the lifetime is critically limited. If doxorubicin is one of the rare candidates of effective agents to treat such as cancer stem cells, the development of drug delivery systems to avoid the side effects should seriously be important.

### 2.2. Expression of MMP-2 in U251MG-P1 Cells

**Figure 2:**
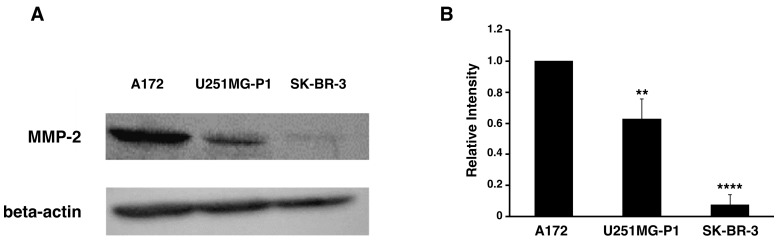

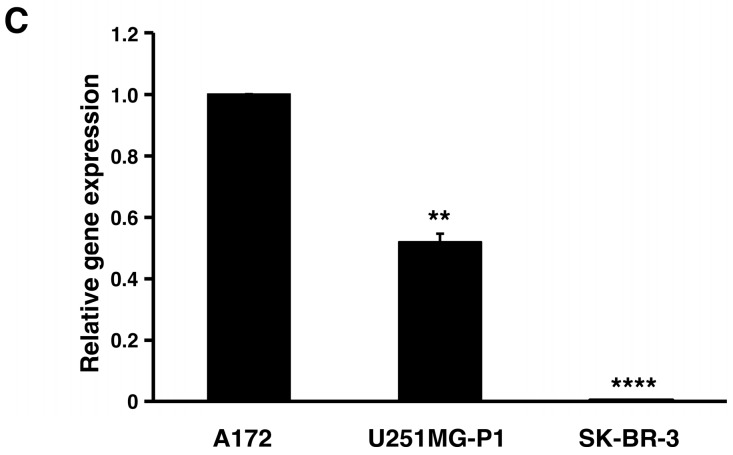
The U251MG-P1 cells are expressing MMP-2. (**A**) Western blot of cells with anti-MMP-2 and anti-beta-actin antibodies. (**B**) Relative intensity of the bands in Western blot densitometrically analyzed by ImageJ. (**C**) Relative gene expression analyzed by reverse transcription quantitative PCR. The data presented as the mean ± SD (*n* = 3) from three independent experiments. The data were analyzed by two-tailed students *t*-test using A172 cells as a control; **, *p* < 0.01; ****, *p* < 0.001.

We assessed the expression of MMP-2 in U251MG-P1 cells to confirm that MMP-2 could be a sufficient marker to target the cells. A172 (MMP-2-positive) and human breast cancer SK-BR-3 (MMP-2-negative) cell line by both Western blot and reverse transcription-polymerase chain reaction (qRT-PCR) as shown in Figure 2A–C. The 72-kDa protein treated with anti-MMP-2 antibody corresponding to proMMP-2 was observed in U251MG-P1 and A172 but less (or not seen) in SK-BR-3. Since U251MG-P1 cells were confirmed to express MMP-2, we decided to employ CTX to target U251MG-P1 cells.

### 2.3. Characterization of M-CTX-Fc

Peptide ligands specific to cell surface molecules have extensively been used for various drug delivery targeting in cancer cells. However, they are often labile and degraded resulting in short half-life due to their antigenicity and reticuloendothelial system (RES) [23]. One of the approaches to overcome those problems is to fuse the ligand with human IgG Fc domain. The Fc domain provides significant advantages such as improving the solubility and stability of partner molecules and to prolong the half-life in plasma [24,25]. 

**Figure 3:**
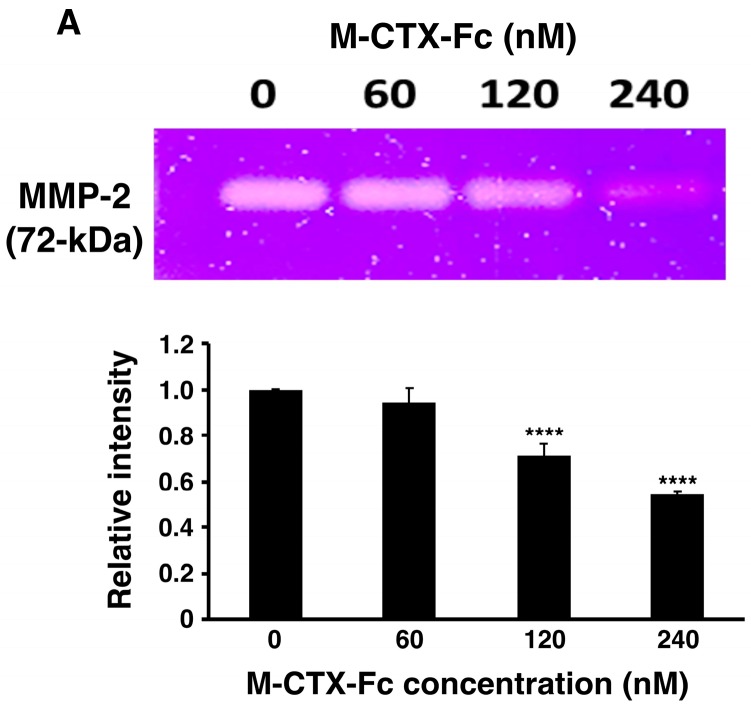

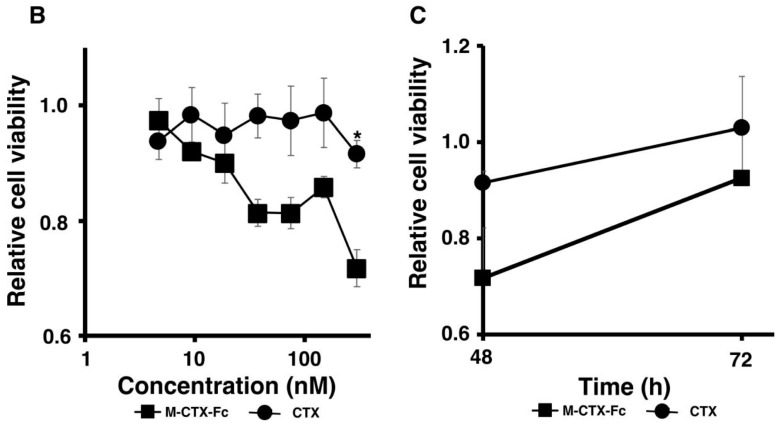
M-CTX-Fc inhibits gelatinase activity in the condition medium of U251MG-P1 cells (**A**) and cell growth of U251MG-P1 cells (**B**,**C**). The M-CTX-Fc inhibit gelatinase activity. (**A**) MMP-2 activity in the condition medium of U251MG-P1 cells was monitored by zymography in the presence of 0, 60, 120, and 240 nM of M-CTX-Fc. The data presented as the mean ± SD (*n* = 3) from technical replicates. The statistical significance in mean values of more than two groups was determined using one-way analysis of variance (ANOVA) and post hoc Tukey HSD were applied using CM without M-CTX-Fc as control *, *p* < 0.05; ****, *p* < 0.001. (**B**) The inhibition of cell growth in the presence of M-CTX-Fc and CTX after 48 h. (**C**) The viable cells at 48 h were kept cultured without M-CTX-Fcs or CTX up to 72 h. Cell numbers in each well were assessed by MTT assay. The absorbance at 570 nm corresponding to the initial number of the cells was defined as 1. The data presented as the mean ± SD (*n* = 3) from three independent experiments. The data were analyzed by 2-tailed students t-test using M-CTX-Fc as a control; *, *p* < 0.05; ****, *p* < 0.001.

After preparation of M-CTX-Fc from *E. coli*, the ability to inhibit the gelatinase activity of secreted MMP-2 in the condition medium of U251MG-P1 cells was observed by gelatin zymography (Figure 3A). The intensity of gelatinase activity decreased in the presence of M-CTX-Fc in a dose-dependent manner. This confirmed the interaction of M-CTX-Fc with MMP-2 even though the exact molecular target of CTX is still unknown [26]. We then evaluated the activity of M-CTX-Fc on the proliferation and viability of U251MG-P1 cells. M-CTX-Fc suppressed the growth of the cells as described in other cells such as the glioma cell line A172 and pancreatic carcinoma cell line PANC-1 cells [16,17] (Figure 3B,C). This cell growth inhibition does not appear to induce cell death because the cell viability recovered after removal of the protein from the culture medium while CTX alone did not affect the cell growth of U251MG-P1. Collectively, we concluded that M-CTX-Fc is folded properly. Hence, we chose M-CTX-Fc instead of CTX as the ligand to target U251MG-P1 cells considering that the Fc moiety would help the conformation of intact CTX when conjugated on the surface of liposomes. 

### 2.4. Characterization and Optimization of M-CTX-Fc Conjugated to Liposome

**Figure 4:**
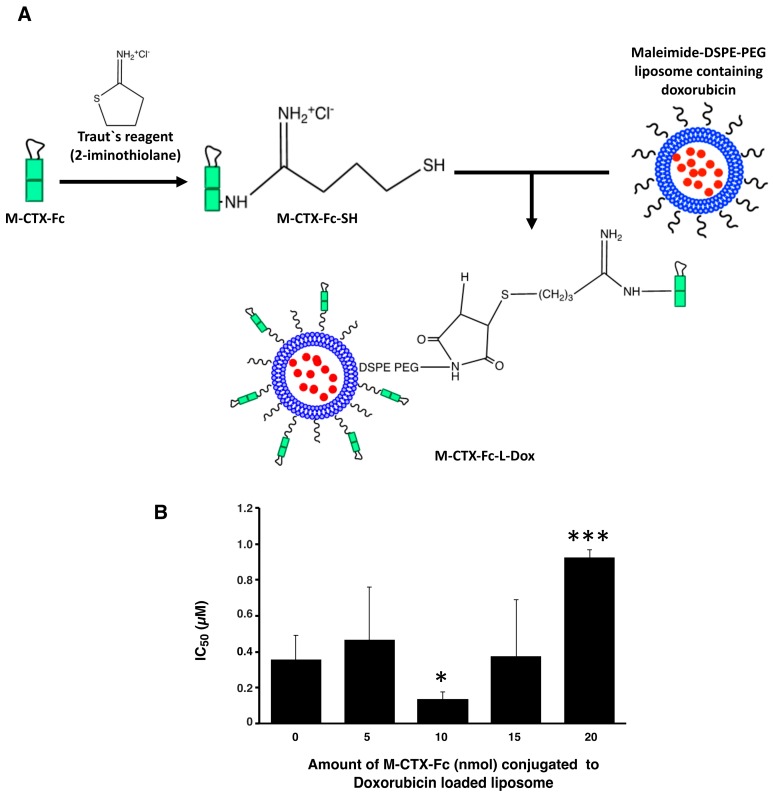
The amount of M-CTX-Fc conjugated to liposomes encapsulating doxorubicin was optimal at 10 nmol/48 µmol DPPC. (**A**) Conjugation procedure of M-CTX-Fc to liposomes encapsulating doxorubicin. (**B**) IC_50_ of doxorubicin encapsulated in liposomes conjugating various amount of M-CTX-Fc) against U251MG-P1 cells. The data presented as the mean ± SD (*n* = 3) from independent experiments. The statistical significance in mean values of more than two groups was determined using one-way analysis of variance (ANOVA) and post hoc Tukey HSD were applied using no M-CTX-Fc (0 mol) as control. *, *p* < 0.05; ***, *p* < 0.005.

Prior to further investigation of their cytotoxicity in vitro and in vivo, we optimized the amount of M-CTX-Fc (nmol) conjugated to liposomes. The preparation of liposomes conjugated with M-CTX-Fc encapsulating doxorubicin (M-CTX-Fc-L-Dox) is summarized in Figure 4A. Various amounts of M-CTX-Fc such as 5, 10, 15 or 20 nmol conjugated to liposome encapsulating doxorubicin was prepared, respectively. The mean particle size of these liposomes was almost 150 nm, which was not significantly affected by the amount of ligand as previously described [27,28]. The optimal amount of M-CTX-Fc conjugated to liposomes was determined by the IC_50_ (Figure 4B). When 10 nmol of M-CTX-Fc were used to conjugate to liposome, the cytotoxicity of doxorubicin was at a maximum whereas 5 nmol was not enough for binding to the receptor. Similarly, M-CTX-Fc at 15 nmol and 20 nmol did not improve the cytotoxic effect of liposome encapsulating doxorubicin. We further investigated the capability of specific targeting of liposomes by using M-CTX-Fc at 10 nmol.

The characteristics of the formulations of liposomes encapsulating doxorubicin used in this study are summarized in Table 1. The liposomes conjugated with human IgG (hIgG-L-Dox) or without ligands (L-Dox) were prepared as references of nonspecific targeting. All the prepared liposomes showed diameters of approximately 150 nm with a low polydispersity index less than 0.1 indicating homogeneous uniformity. The uniformity of particle size is considered important to obtain stable receptor-mediated endocytosis in the intracellular delivery by the nano-carrier system [29]. A particle size between 50 and 200 nm is considered sufficient to accumulate the drug in the tumor via enhanced permeability and retention (EPR) effect since particles with larger sizes are generally trapped by the RES resulting in a short half-life by rapid clearance from blood flow [30]. 

**Figure 5:**
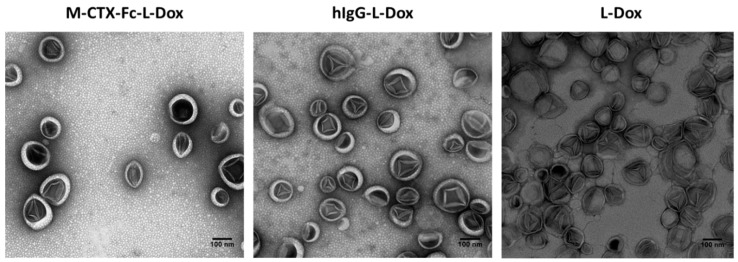
M-CTX-Fc-L-Dox showed unilamellar vesicles with diameter of approximately 100 nm. TEM images of liposome formulation encapsulating doxorubicin were compared between M-CTX-Fc-L-Dox, hIgG-L-Dox and L-Dox. Each scale bar shows 100 nm.

Transmission electron microscopy (TEM) revealed that all the formulations of liposome encapsulating doxorubicin exhibited precipitates of fibrous-bundle aggregates when doxorubicin was encapsulated into the inner core of liposomes due to the loading method of pH gradient (Figure 5) [19].

### 2.5. Cellular Uptake of Liposomes

**Figure 6:**
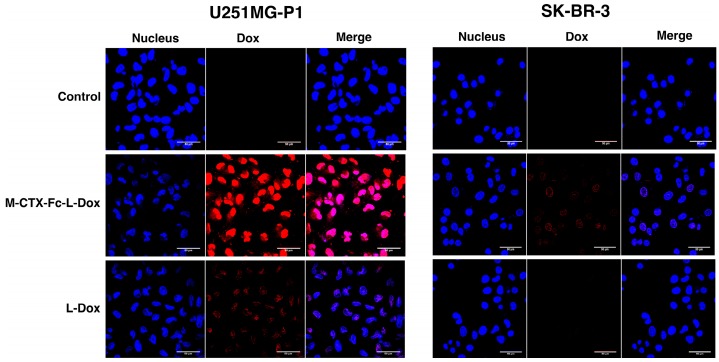
Cellular uptake of Doxorubicin in U251MG-P1 cells was enhanced through M-CTX-Fc-L-Dox. U251MG-P1 cells and SK-BR-3 cells were evaluated for the cellular uptake of doxorubicin under a confocal microscope. Cell nuclei were stained with DAPI (blue). Red color arises from natural fluorescence properties of doxorubicin. Each scale bar shows 50 µm.

**Figure 7:**
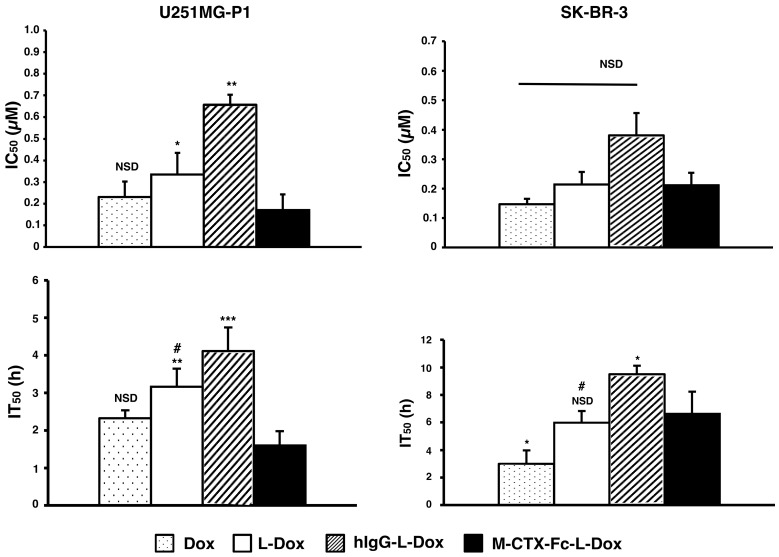
M-CTX-Fc-L-Dox exhibited the lowest inhibition concentration (IC_50_) and shortest exposure time (IT_50_) in U251MG-P1 cells. In vitro cytotoxicity IC_50_ of doxorubicin in different formulations after 72 h of exposure to U251MG-P1 and SK-BR-3 cells was evaluated and compared (top). IT_50_ with doxorubicin at IC_100_ were evaluated and compared (bottom). The data presented as the mean ± SD (*n* = 3). The statistical significance in mean values of more than two groups was determined using one-way analysis of variance (ANOVA) and post hoc Tukey HSD were applied using M-CTX-Fc-L-Dox liposome as control. *, *p* < 0.05; **, *p* < 0.01; ***, *p* < 0.005; NSD, no significant difference; #, *p* < 0.05 versus Dox.

The cellular uptake of M-CTX-Fc-L-Dox and L-Dox into U251MG-P1 and SK-BR-3 cells was evaluated under a confocal microscope after 1 h incubation at 37 °C (Figure 6). It is worth noting that the strong fluorescence of doxorubicin was observed in U251MG-P1 cells treated with M-CTX-Fc-L-Dox, especially in the nuclei. This observation might be attributed to the specific interaction of M-CTX-Fc-L-Dox with the cell surface molecule on U251MG-P1 cells by receptor-mediated endocytosis. On the other hand, when cells were treated with L-Dox, the fluorescence from L -Dox was reduced compared to that from M-CTX-Fc-L-Dox. It is important to mention that receptor-mediated endocytosis achieved by M-CTX-Fc-L-Dox might be faster than endocytosis gained by L-Dox only. This explanation agrees with our result for IT_50_ in Figure 7. Oppositely, almost no signal for doxorubicin uptake was observed in the SK-BR-3 cells, which showed low expression of MMP-2 compared to U251MG-P1 cells. Collectively, M-CTX-Fc may have the potential to target MMP-2-expressing cancer cells and internalize into the cells while further investigation is required to identify the molecule on the cell surface directly binding to M-CTX-Fc. 

### 2.6. Cytotoxicity In Vitro

The IC_50_s of doxorubicin were assessed when U251MG-P1 and SK-BR-3 cells were treated for 72 h with each formulation of naked doxorubicin, L-Dox, M-CTX-Fc-L-Dox and hIgG-L-Dox (Figure 7). Among the liposomal formulations, M-CTX-Fc-L-Dox showed the highest cytotoxicity with the lowest IC_50_ of 0.17 µM in U251MG-P1 cells. However, the cytotoxicity of M-CTX-Fc-L-Dox showed no significant different with naked doxorubicin. In SKBR-3 cells without MMP-2 expression, M-CTX-Fc-L-Dox appeared almost equally effective with hIgG-L-Dox and L-Dox. Collectively, the results appear consistent with the dependency of MMP-2 expression. As described previously, we thought the time of exposure allowing the cellular uptake should also be important to determine the effectiveness [31]. To make this point clearer, we evaluated the IT_50_. 

In U251MG-P1 cells, M-CTX-Fc-L-Dox showed significantly rapid exposure time of IT_50_ at around 1.6 h. This is the shortest time when compared with those by other formulations. Meanwhile, in both cells, naked doxorubicin had rapid exposure time compared to L-Dox. This result could be explained by the difference of cellular mechanism of internalization. The cellular uptake of liposomes is mediated by endocytosis, whereas the naked doxorubicin molecules internalized into the cell via passive diffusion. However, in the case of M-CTX-Fc-L-Dox, the conjugated ligand specific to MMP-2 receptor, exhibited shorter time for liposomes internalized into the cells and reaches IT_50_ comparable to the naked doxorubicin. On the other hand, in SK-BR-3 cells, no significant difference was found between L-Dox and M-CTX-Fc-L-Dox. Thus, M-CTX-Fc-L-Dox successfully demonstrated the specific targeting of U251MG-P1 cells in vitro.

### 2.7. Suppression of Tumor Growth In Vivo

**Figure 8:**
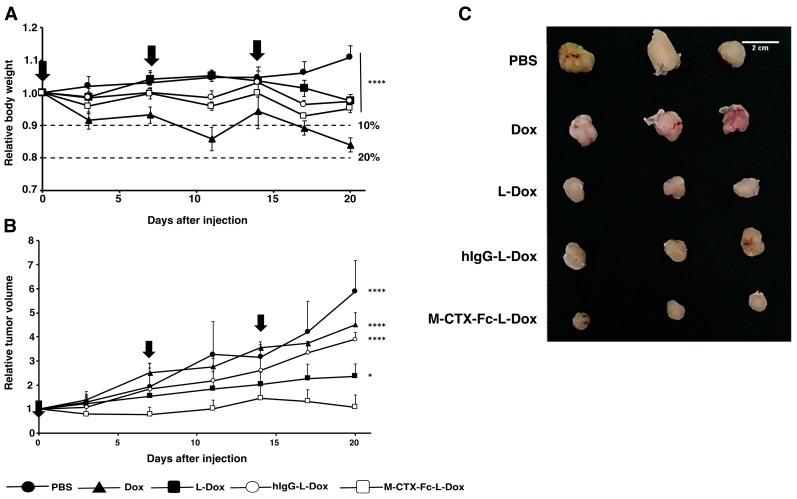
M-CTX-Fc-L-Dox suppressed tumor growth in the most effective manner in vivo. (**A**) The relative body weight of mice bearing tumors during the treatment. M-CTX-FC-L-Dox and other liposome formulations were less toxic than naked doxorubicin. The statistical significance in mean values of more than two groups was determined using one-way analysis of variance (ANOVA) and post hoc Tukey HSD were applied using relatives body weight of Dox treatment as control. ****, *p* < 0.001. (**B**) The effect of different formulations of doxorubicin on the volume of tumors. M-CTX-Fc-L-Dox was the most effective formulation to suppress the growth of tumor. Doxorubicin in each formulation was administered at 7-day intervals (indicated by vertical arrows). The statistical significance in mean values of more than two groups was determined using one-way analysis of variance (ANOVA) and post hoc Tukey HSD were applied using relatives tumor volume of M-CTX-Fc-L-Dox treatment as control, *, *p* < 0.05; ****, *p* < 0.001 (**C**) The tumors from the experiment (**B**) representing each group were displayed exhibiting the effect of each formulation of doxorubicin. Data are expressed as the mean with ±SD where *n* = 3.

The suppression of tumor growth by M-CTX-Fc-L-Dox was evaluated in BALB/c mice bearing tumors of transplanted U251MG-P1 cells (Figure 8). We found that the tumor latency of U251MG-P1 is so rapid that it should not be comparable with that of U251MG cells. Meanwhile, the tumor latency of U251MG is not stable. This means that the targeting effect of our liposomal formulation is difficult to be demonstrated on the tumors from U251MG cells. First of all, the effect of doxorubicin on the body weight was assessed by the three-time injections of 10 mg/kg (Figure 8A). As a result, the loss of body weight was less than 20% even when the naked doxorubicin was injected. The liposomal formulations were less toxic than naked doxorubicin as they showed body weight loss less than 10%. After three times of injection in seven-day intervals, the tumor growth was observed for 20 days, and the efficacy of the suppression of tumor growth was calculated as a relative tumor volume normalized to the initial tumor volume before the treatment. The tumor volume in the PBS group increased aggressively, whereas M-CTX-Fc-L-Dox slowed the tumor growth more significant than naked Dox and hIgG-L-Dox at day 20 (*p* < 0.001) (Figure 8B). M-CTX-Fc-L-Dox appeared slightly more effective than L-Dox at day 20 (*p* = 0.043 < 0.05). The representative tumors excised from mice treated with five different formulations at day 20 demonstrated tumor suppression effect of M-CTX-Fc-L-Dox (Figure 8C). In this context, M-CTX-Fc-Dox exhibited an inhibitory effect on tumor growth, which could be attributed to the combined action of the passive targeting via the EPR effect and active targeting via receptor-mediated endocytosis. However, a larger cohort-evaluation is needed to confirm the effect of M-CTX-Fc-L-Dox more precisely.

## 3. Materials and Methods

### 3.1. Materials

Dipalmitoylphosphatidylcholine (DPPC), 1,2-distearoyl-sn-glycerol-3 phosphoethanolamine-*N*-[methoxy (polyethylene glycol)-2000] (mPEG–DSPE) and 1,2-distearoyl-sn-glycerol-3-phosphoethanolamine-*N*-[maleimide (polyethylene glycol)-2000] (Mal–PEG–DSPE) were obtained from NOF Co. (Tokyo, Japan). Cholesterol (Chol) was purchased from Kanto Chemical Co., Inc. (Tokyo, Japan). Thiazolyl blue tetrazolium bromide (MTT), 2-iminothiolane hydrochloride, human IgG, RPMI 1640 medium, and DMEM were from Sigma-Aldrich (St Louis, MO, USA). Doxorubicin hydrochloride was purchased from Wako Pure Chemical (Osaka, Japan). Amicon Ultra filters were from Merck Millipore Ltd. (Billerica, MA, USA). PD-10 columns were from GE Healthcare (Carlsbad, CA, USA).

### 3.2. Cell Cultures and Experimental Animals

The human glioblastoma cell line A172 cells and human breast cancer cell line SK-BR-3 cells were obtained from ATCC (Manassas, VA, USA). U251MG-P1 was isolated from a xenograft tumor of human glioblastoma cell line U251MG cells in mouse [18]. A172 and U251MG-P1 were maintained in DMEM medium supplemented with 10% fetal bovine serum (FBS) (PAA Laboratories, Pasching, Austria) in the presence of penicillin (100 IU/mL) and streptomycin (100 μg/mL) (Nacalai Tesque, Kyoto, Japan). SK-BR-3 cells were cultured in RPMI 1640 medium supplemented with 10% FBS in the presence of 100 IU/mL penicillin and 100 μg/mL streptomycin. Cells were cultured in a humidified incubator at 37 °C with the atmosphere of 5% CO_2_.

Four-week-old female BALB/c nude mice from Charles River (Kanagawa, Japan) were bred at 23 °C and fed with sterilized food and water during the experiments. All animal experimental protocols were reviewed and approved by the ethics committee (Animal Care and Use Committee) of Okayama University under the project identification code IDs OKU-2016078 (Date of approval: 1 April 2016).

### 3.3. Preparation of M-CTX-Fc

M-CTX-Fc fusion protein was produced in our laboratory by recombinant expression in *E. coli* [14,15]. Briefly, M-CTX-Fc was expressed in *E. coli* as inclusion body and was refolded (Figure S1 in Supplementary Material). 

### 3.4. Gelatin Zymography for MMP-2 Activity

MMP-2 gelatinolytic activity in the CM was determined by zymography. Fifteen microliter aliquots of CM with or without M-CTX-Fc were subjected to 10% SDS-PAGE containing 0.05% gelatine. The samples were prepared without reducing reagent and boiling prior to electrophoresis. After electrophoresis, the gel was washed twice in 2.5% Triton X-100 for 30 min and once in 50 mM Tris-HCI, pH 7.4, 10 mM CaCl_2_ and 0.02% NaN_3_. After incubation, gel was stained with Coomassie brilliant blue in 50% methanol and 10% acetic acid and destained in 10% methanol and 10% acetic acid.

### 3.5. Preparation of Liposomes Encapsulating Doxorubicin

#### 3.5.1. Encapsulation of Doxorubicin into Liposomes

Liposomes composed of DPPC and Chol with 5 mol % mPEG–DSPE were prepared by the thin-film hydration method followed by the transmembrane pH gradient method [19,32]. In brief, DPPC, Chol, and mPEG–DSPE were dissolved in an organic solvent mixture consisting of chloroform and methanol (9:1, *v*/*v*) in a round-bottom flask equipped with rotary evaporator at 50 °C under aspirator vacuum. The resulting lipid film was left overnight under vacuum to ensure that all traces of organic solvent are removed from the film. Then, the film was hydrated with 300 mM citrate buffer, pH 4.0, by gentle mixing, resulting in spontaneously organized multilamellar vesicles (MLVs). MLVs were freeze-thawed five times and passed through a Whatman polycarbonate membrane with a pore size of 100 nm (GE Healthcare, Carlsbad, CA, USA) ten times using an extruder (Avanti Polar Lipids, Inc., Alabaster, AL, USA) to form small, unilamellar vesicles. The liposome suspension was eluted using Sephadex G-25 (PD-10 desalting column) pre-equilibrated with PBS, pH 7.4, to form a pH gradient. Dox at pH 7.4 was introduced into the liposome suspension, and an excess of free Dox was removed by washing with PBS followed by ultrafiltration using a 100K-membrane filter.

#### 3.5.2. Preparation of M-CTX-Fc-L-Dox or hIgG-L-Dox

The M-CTX-Fc or human IgG were coupling to liposome using classical method by employing the maleimide-thiol addition reaction [33]. Briefly, 2 mol % of Mal–PEG–DSPE were incubated with Dox loaded liposome at 50 °C for 10 min. Simultaneously, thiol group (-SH) were introduced into M-CTX-Fc and human IgG by incubation with Traut’s reagent (2-iminothiolane) at molar ratio of 1:10 and 1:50 in 25 mM HEPES, pH 8.0, containing 140 mM NaCl respectively. The reaction occurred under gentle stirring for 1 h in the dark at room temperature. Unreacted 2-IT reagent was removed by using gel chromatography G25 PD-10 column (GE Healthcare, Carlsbad, CA, USA). Thiolated M-CTX-Fc or human IgG was then coupled to Mal–PEG–DSPE by thioether linkage. The coupling reaction was performed overnight in the dark with gentle stirring at 4 °C. Free M-CTX-Fc or human hIgG was removed by ultrafiltration with 100K and 300K membrane filter respectively (Sartorius Stedim Biotech GmbH, Goettingen, Germany) (Figure S2).

### 3.6. Characterization of Liposomes

#### 3.6.1. Size Distribution and Zeta Potential

The size and zeta potential of liposomes were determined by dynamic and electrophoretic light scattering using an ELS-8000 (Photal Otsuka Electronics, Osaka, Japan). 

#### 3.6.2. Encapsulation Efficiency (EE) and Loading Efficiency (LE)

The concentration of doxorubicin was quantified by microplate reader SH-9000 (Corona Electric, Ibaraki, Japan) at 490 nm. EE was then calculated as the amount of drug loaded in the liposomes divided by initial amount of the drug. LE was calculated as the molar ratio of the drug loaded into liposomes to the total of lipid and Chol.

### 3.7. Evaluation of Cellular Uptake of Liposomes 

The cellular uptake of liposomes was evaluated according to the previously described method [15]. U251MG-P1 and SK-BR-3 cells were cultured on gelatin-coated glass coverslips in a 12-well plate at a density of 3 × 10^5^ cells per well for 24 h. Then, M-CTX-Fc and L-Dox (containing Dox at 30 µg/mL) were added to a serum-free medium and incubated at 37 °C in the darkness for 1 h. The cells treated with a medium were used as a negative control. After the incubation, cells were washed three times with cold PBS and fixed with 4% paraformaldehyde for 20 min. After nuclear staining with DAPI (Vector Laboratories, Burlingame, CA, USA), the fluorescent signal was imaged using a laser scanning confocal microscope (FV-1000, Olympus, Tokyo, Japan).

### 3.8. Cytotoxic Assay

To investigate the in vitro cytotoxicity of various Dox-loaded liposomes in U251MG-P1 and SK-BR-3 cells, tetrazolium reduction assay was employed as previously described [31]. Briefly, 5000 cells/well were seeded onto a 96-well plate and cultured for 24 h. Then, the cells were incubated with 50 µL of a culture medium containing free doxorubicin or different liposome formulations with various doxorubicin concentrations for 72 h at 37 °C and 5% CO_2_. Afterward, the cells were exposed to 5 mg/mL of MTT in PBS (the final concentration of 1 mg/mL) for 4 h. Formazan crystals formed during the incubation period were dissolved overnight at 37 °C by adding 10% SDS containing 20 mM HCl. The absorbance was measured at 570 nm using 96-well microplate reader SH-9000 (Corona Electric, Ibaraki, Japan). The exposure time required to kill 50% of the cells was evaluated as IT_50_, which should be obtained at the minimum concentration of doxorubicin killing 100% of the cells after 72 h of exposure defined as IC_100_. 

### 3.9. Time-Dependent Cytotoxic Effects

Time-Dependent cellular cytotoxicity [31] was evaluated by the MTT assay with drugs at IC_100_ (Supplementary Table S1). Briefly, U251MG-P1 and SK-BR-3 were seeded in a 96-well plate at 5 × 10^3^ cell/well. After incubation at 37 °C under 5% CO_2_ for 24 h, drugs at each IC_100_ were added to each well and incubated for 1, 2, 6, 12, 24, 48, and 72 h. After each round of drug exposure, the medium was replaced with fresh medium without drugs and the incubation was continued until 72 h. Cell viability was determined by MTT assay. The time required for 50% growth inhibition (IT_50_) was estimated from the survival curve.

### 3.10. Anti-Tumor Study In Vivo

The xenograft of U251MG-P1 cells in mice was prepared by a subcutaneous injection of 1 × 10^6^ cells/mouse. Tumor volume was measured by a vernier caliper and calculated as [length × (width)^2^]/2. Anti-tumor effect of each formulation was evaluated when the tumor volume reached 100–200 mm^3^. Mice were randomly assigned to five groups (*n* = 3); group 1 for saline, group 2 for naked Dox, group 3 for L-Dox, group 4 for hIgG-L-Dox and group 5 for M-CTX-Fc-L-Dox. Ten mg of doxorubicin per kg body weight was injected three times via tail vein at the intervals of 7-day. Tumor volume was measured every three days.

### 3.11. Statistical Analysis

All the experiments were at least three-time repetition. Data were depicted as means ± standard deviation. The statistical significance in mean values between two groups was determined by 2-tailed student’s *t*-test. The statistical significance between the mean values of more than two groups was determined using one-way analysis of variance (ANOVA) and post hoc Tukey HSD. A *p*-value less than 0.05 was considered to be statistically significant, and a *p*-value less than 0.01 was regarded as highly significant.

## 4. Conclusions

In our previous study, U251MG-P1 cells had shown CD44 expression was enriched when compared to the parental U251MG cells, along with aberrant activation of principal stemness marker genes *OCT3/4*, *SOX2*, *KLF4* and *Nanog* and with less expression of glial fibrillary acidic protein. This meant that U251MG-P1 cells consist of rather undifferentiated cells than the parental U251MG cells. Additionally, U251MG-P1 cells are highly tumorigenic exhibiting rapid tumor growth in vivo, when compared to U251MG cells. This high tumorigenicity enabled us to study the drug delivery in vivo.

Drug delivery targeting cancer cells derived from glioblastoma was successfully demonstrated in vitro and in vivo with chlorotoxin fused to human IgG Fc domain in this study. The results showed that chlorotoxin fusion protein shortened the IT_50_ of doxorubicin encapsulated in liposomes in vitro and suppressed the growth of tumor in vivo when compared with the liposomes without chlorotoxin ligand. Although we could not show the direct binding of M-CTX-Fc to MMP-2, we could show significant difference in the relative tumor growth between the treatment with M-CTX-Fc-L-Dox and that with L-Dox at day 20 (*p* < 0.05). This difference appeared to depend on the expression of MMP-2 according to a previous report, which demonstrated the specific interaction of chlorotoxin and MMP-2 [10]. Thus, the combination of doxorubicin and chlorotoxin is proposed in this paper as a successful candidate of liposomal DDS formulation to target glioblastoma cells and derivative tumors.

## Figures and Tables

**Table 1:** Characteristics of the formulations of liposomes encapsulating doxorubicin.

Formulations	Diameter (nm)	Polydispersity Index	Zeta Potential (−mV)	Encapsulation Efficiency (%)	Loading Efficiency (%)
L-Dox	133.4 ± 12.7	0.09 ± 0.03	8.13 ± 2.32	97.5 ± 3.1	3.4 ± 0.1
M-CTX-Fc-L-Dox	148.3 ± 3.0	0.05 ± 0.03	7.86 ± 1.19	98.2 ± 1.3	4.5 ± 0.4
hIgG-L-Dox	151.3 ± 4.3	0.07 ± 0.02	6.66 ± 3.78	94.4 ± 7.2	4.1 ± 0.4

Each experiment was performed in triplicate and the values are given as mean ± SD.

## Supplementary Materials

The following figures are available online at http://www.mdpi.com/1422-0067/19/3/659/s1.

## Abbreviations

BBB	Blood brain barrier
MMP-2	Matrix metalloproteinase-2
CTX	Chlorotoxin
DPPC	Dipalmitoylphosphatidylcholine
MTT	Thiazolyl blue tetrazolium bromide
RES	Reticuloendothelial system
EPR	Enhanced permeability retention
TEM	Transmission electron microscopy
*E. coli*	*Escherichia coli*
FBS	Fetal bovine serum
M-CTX-Fc-L-Dox	Liposomes conjugated with M-CTX-Fc encapsulating doxorubicin
hIgG-L-Dox	Liposome conjugated with human IgG encapsulating doxorubicin
L-Dox	Liposome encapsulating doxorubicin without ligand
