# Supplementary material for: Targeting Glioblastoma Cells Expressing CD44 with Liposomes Encapsulating Doxorubicin and Displaying Chlorotoxin-IgG Fc Fusion Protein
Source: Int J Mol Sci. 2018 Feb 26;19(3):659. doi: 10.3390/ijms19030659 (PMC5877520; doi:10.3390/ijms19030659)
Supplement: Supplementary file 1 [file ijms-19-00659-s001.pdf]

Supplementary Documents

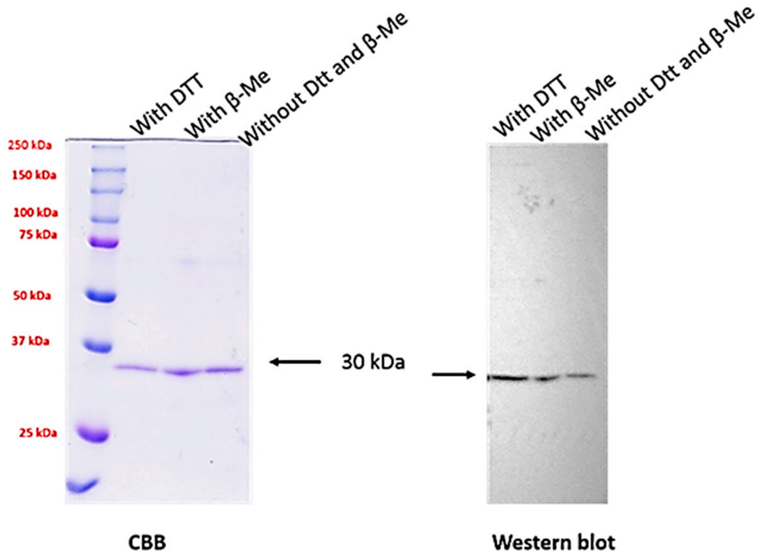

Figure S1. M-CTX-Fc in reducing and nonreducing conditions. Purified protein was subjected to SDS-PAGE and detected by CBB staining (left) and Western blotting with mouse monoclonal anti-human IgG antibody (right) as monomer at approximately 30 kDa.

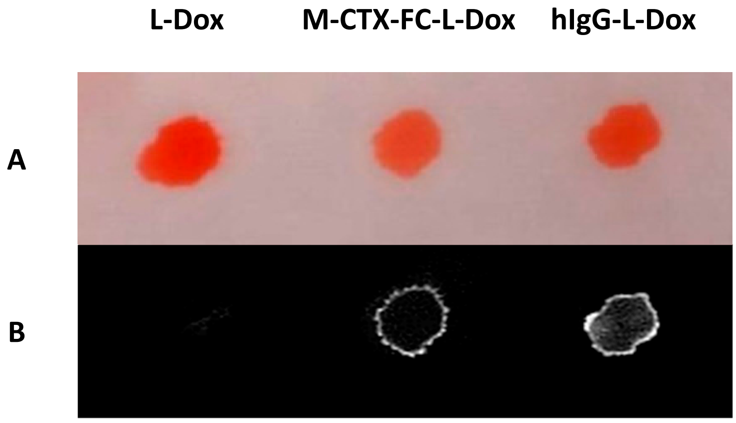

Figure S2. Dot blotting analysis of liposomes conjugated to ligands. Liposomes containing approximately 1 μg of doxorubicin in 3 μL were blotted onto PVDF membrane (**A**) and probed with mouse monoclonal anti-human IgG antibody conjugated with HRP (**B**). The immunoreactivity indicates the succesful conjugation of M-CTX-Fc and hIgG to liposomes.

Table S1. Cytotoxicity of different Dox formulations in U251MG-P1 and SK-BR-3.

	U251MG-P1			SK-BR-3		
	**IC$_{50}$ (μM)**	**IC$_{100}$ (μM)**	**IT$_{50}$ (h)**	**IC$_{50}$ (μM)**	**IC$_{100}$ (μM)**	**IT$_{50}$ (h)**
Dox	0.19 ± 0.11	1	2.3 ± 0.2	0.15 ± 0.02	1	3.0 ± 1.0
L-Dox	0.35 ± 0.08	5	3.4 ± 0.4	0.21 ± 0.05	1	6.0 ± 0.8
hIgG-L-Dox	0.66 ± 0.05	10	4.1 ± 0.6	0.38 ± 0.06	1	9.5 ± 0.6
M-CTX-Fc-L-Dox	0.17 ± 0.07	1	1.6 ± 0.4	0.21 ± 0.04	1	6.6 ± 1.6

IC50 and IT50 are presented as the mean ± S.D. (n = 3). IC100 was estimated from the evaluation of cytotoxicity.
